# Sintilimab-induced erythema multiforme drug eruption in the treatment of sigmoid colon cancer: A case report and literature review

**DOI:** 10.1097/MD.0000000000035659

**Published:** 2023-10-13

**Authors:** Mei Zhang, Ran Wu, Min Jia, Shaoqin Sun, Lei Zhang, Ting Tang

**Affiliations:** a Guizhou University of Traditional Chinese Medicine, Guiyang, China; b Department of Dermatology, The First Affiliated Hospital of Guizhou University of Traditional Chinese Medicine, Guiyang, China; c Department of Dermatology, The Second People’s Hospital of Guiyang, Guiyang, China.

**Keywords:** case report, erythema multiforme drug eruption, immune-related cutaneous adverse events (ircAEs), PD-1 inhibitors, sintilimab

## Abstract

**Rationale:**

Dermatologic toxicity has been reported as the most common immune-related side effect of programmed cell death 1 inhibitors. Previous reports related to Sintilimab include rash, pruritus, vitiligo, Stevens-Johnson syndrome, toxic epidermal necrolysis, and so on.

**Patient concerns:**

A 66-year-old man was treated with Sintilimab as monotherapy for sigmoid colon cancer. After the second prescription, he developed a more severe and widespread rash.

**Diagnoses:**

The diagnose of erythema multiforme drug eruption induced by Sintilimab was considered.

**Interventions:**

The patient received intravenous and oral methylprednisolone, routine antihistamines and topical gluccorticoids.

**Outcomes:**

The patient’s symptoms were gradually relieved during hospitalization and was discharged following resolution of symptoms. He refused to continue using Sintilimab.

**Lessons:**

This is the first reported case of Sintilimab-induced erythema multiforme drug eruption. It is advisable to inform patients of potential dermatologic toxicity that may occur after using immune checkpoint inhibitors, so that we may prevent the further development of it and avoid the discontinuation of immune checkpoint inhibitors.

## 1. Introduction

Sintilimab is a recombinant fully human anti-programmed cell death protein 1 monoclonal antibody that was independently developed in China, which belongs to 1 kind of immune checkpoint inhibitors (ICIs). Erythema multiforme (EM) drug eruption is a comperatively common cutaneous drug reaction. Here, we describe a 66-year-old man who was treated with Sintilimab after resection for sigmoid colon cancer as the only treatment. After 2 days of first administration, the patient developed localized rash. After second prescription of Sintilimab, diffuse rash can be seen all over the body with erosion and crust on his mucosa of labial and external urethral orifice. Based on the patient’s medical history, clinical manifestations and assistant examinations, the final diagnose of EM drug eruption was made. Intravenous methylprednisolone was initially administered, followed by oral methylprednisolone. His rash gradually resolved during hospitalization and was discharged following resolution of symptoms. Sintilimab has not been used since the follow-up. To our knowledge, this kind of adverse event which caused by Sintilimab has not been reported previously. In addition, we briefly discussed the relationship between immune-related side effects and efficacy of ICIs, and whether ICIs can be restarted. We reviewed existing literature on Sintilimab Sintilimab-induced cutaneous adverse events as well.

## 2. Case report

We report the case of a 66-year-old male patient who had a history of sigmoid colon cancer. On March 17, 2022, the patient came to our hospital with multiple pinhead-sized to peanut-sized red to purplish-red macules and maculopapules all over the body, especially in the trunk, with erosion and crust on his mucosa of labial and external urethral orifice. Tracing back his history of present illness: Because of the intolerance to chemotherapy, the patient accepted biologic treatment after resection for sigmoid colon cancer. Two days after the first cycle treatment of Sintilimab (200 mg, intravenously) (Innovent and Lilly Biopharmaceutical company, product and batch number unknown), he found localized red rash in his abdomen and thigh near the groin with mild itching, but symptoms above gradually improved to subsided within a few days without any treatment. Twenty-One days later (21-days cycle intervals), after the second prescribed use of Sintilimab (Innovent and Lilly Biopharmaceutical company, product number: DP2108019, batch number: P2110047), he complained that the similar rash spread to the whole body, as well as with mild itching. Moreover, there were emerging erosion and crust on his mucosa of labial and external urethral orifice. The symptoms did not resolve despite treatment with topical gluccorticoids and oral routine antihistamines. No definite diagnosis was made before his presentation in our hospital.

**Figure 1.**
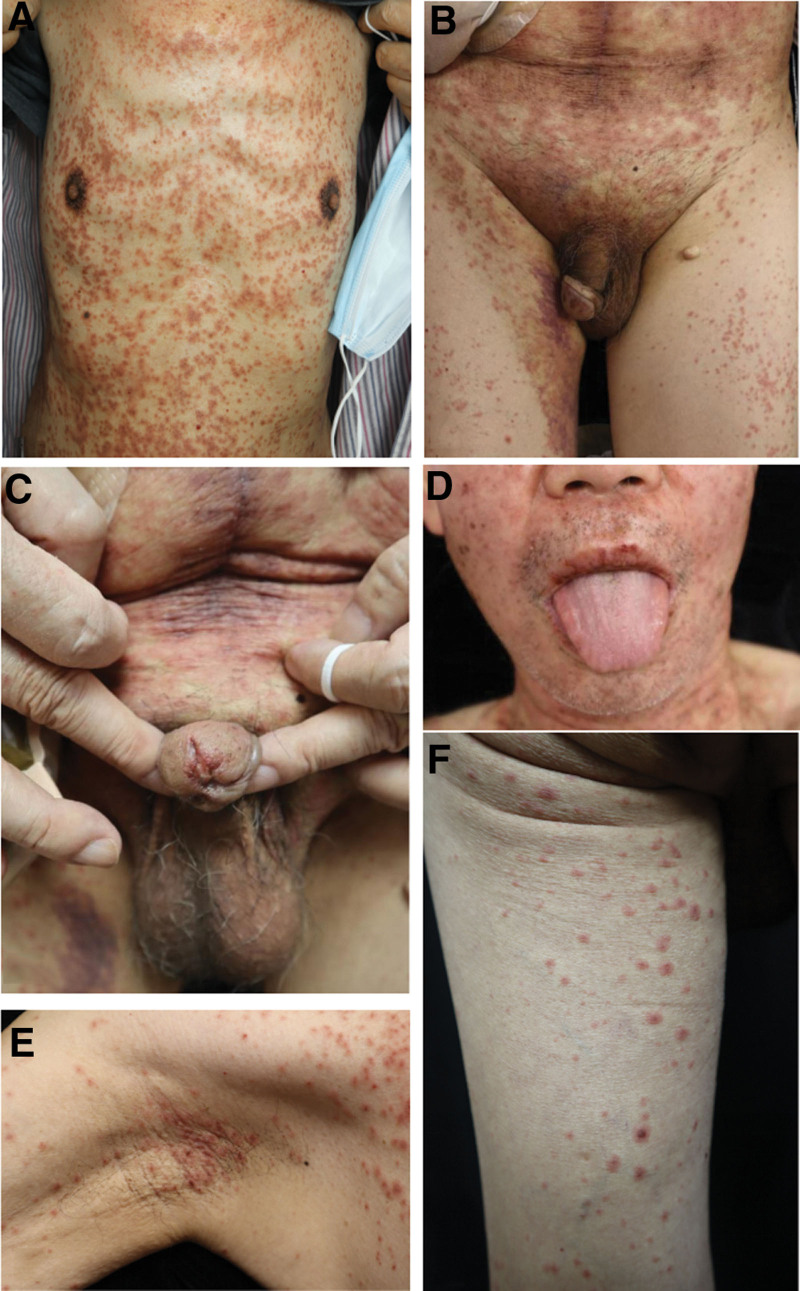
(A–D) Multiple pinhead-sized to peanut-sized red to purplish-red macules and maculopapules can be seen all over the body, especially in the trunk, with erosion and crust on his mucosa of labial and external urethral orifice. (E and F) Typical target-shaped lesions were seen in the flexor side of the thigh and armpit.

On the same day, he was admitted to our hospital with the diagnosis of drug eruption. The patient denied the history of drug and food allergy. Physical examination showed multiple pinhead-sized to peanut-sized red to purplish-red macules and maculopapules were seen all over the body, especially in the trunk, which did not fade when pressed down and varied in density. The macules in the middle and lower abdomen and the upper groin fused into patches, and the macules in the thoracoabdominal were in reticular fusion. Large ecchymosis could be seen in the inner side of the right thigh. Some of the rashes were isolated round-like erythema in mungbean-size to soybean-size, typical target-shaped lesions were seen in the flexor side of the thigh and armpit. Erosion and crust can be seen in the mucosa of labial and external urethral orifice (Fig. 1).

**Figure 2.**
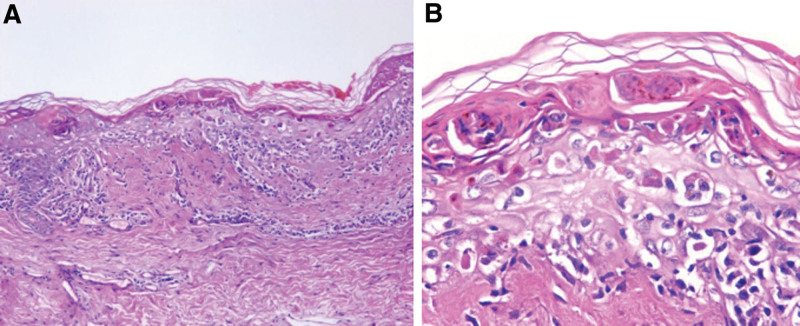
(A and B) Histopathology from an intact erythema on the left costal region shows hyperkeratosis, necrotic keratinocytes could be seen within the epidermis, and there was a tendency to form intraepidermal blisters locally. Liquefaction and degeneration of basal cells, visible interface dermatitis change, and lymphocytic infiltration around blood vessels in the superficial dermis were observed.

A biopsy of a erythema on the left costal region demonstrated histopathological findings of EM (Fig. 2) and the detection of herpes simplex virus, rubella virus, Epstein-Barr virus, cytomegalovirus and human immunodeficiency virus all showed negative. Rash was a known cutaneous adverse event of Sintilimab, no drugs which may cause eruptions were used and no irrational use was found in the course of drug application. The result of Naranjo’s Naranjo probability scale is 8, classified as probable.^[1]^ According to his medical history, clinical manifestations and assistant examinations, we clarify the diagnosis of EM drug eruption.

**Figure 3.**
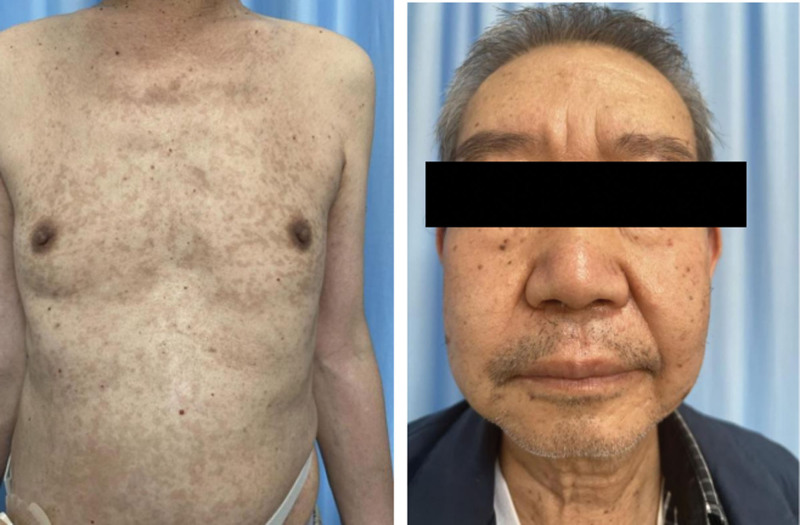
No recurrence of rash was found.

**Figure 4.**
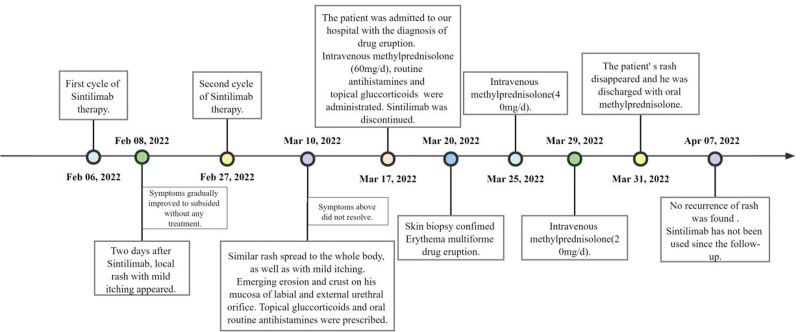
Timeline of diagnosis and major interventions.

We started i. v. administration of 60 mg/days methylprednisolone, combined with routine antihistamines such as Desloratadine Citrate Disodium and topical gluccorticoids after admission. During hospitalization, the patient’s skin lesions gradully improved, and the dose of methylprednisolone was changed to 40 mg/days and finally reduced to 20 mg/days. On the 14th day of hospitalization, the patient was discharged with oral methylprednisolone. On April 7, 2022, he returned to the dermatological clinic of our hospital, and no recurrence of rash was found (Fig. 3). Sintilimab has not been used since the follow-up. The timeline of diagnosis and major interventions is presented in Figure 4.

## 3. Discussion

EM is an acute, immune-mediated reaction that involves the skin and sometimes the mucosa, including the oral cavity. In addition to infectious factors, the second most frequently identified cause of EM is drugs. The clinical manifestations and histopathological findings of EM drug eruption is similar to EM, but the skin lesions of the former are more colorful and more extensive.

ICIs can work by releasing the inhibitory brakes of T cells, resulting in robust activation of the immune system and productive antitumor immune responses, which have revolutionised the management of many cancers.^[2]^ They have been widely used for melanoma, non-small cell cell-lung cancer, renal cancer, and other solid tumors and hematologic malignancies, not only in the metastatic, but also in the adjuvant settings.^[3]^ While ICIs unleash an antitumor T cell response in many patients, enhanced immunologic activation can also cause immune-related adverse events (irAEs) affecting any organ system, we call it immune-related cutaneous adverse events (ircAEs) when involve the cutaneous system.

Sintilimab, a programmed cell death protein 1 inhibitor, belongs to 1 kind of ICIs, which can competitively bind to the PD-1 sites located on T cells, thereby altering the negative regulatory status of T cell-mediated immune responses. Studies have found that^[4]^ the most common irAEs of programmed cell death 1 inhibitors are skin manifestations, accounting for about 30% to 40% of immune-related adverse events, including rash, pruritus, vitiligo, etc. In addition to the above, the severe skin adverse events that may occur after the use of Sintilimab include bullous dermatitis, toxic epidermal necrolysis, exfoliative dermatitis and so on, which can endanger life and deserve attention. By searching the literature, 14 case reports of ircAEs that induced by Sintilimab were found (Table 1).

**Table 1:** Previously reported cases of sintilimab-induced cutaneous adverse events.

	Characteristics	Disease	Therapy regimen	Time to Onset	Cutaneous adverse evevts	Interventions	Stop/restart sintilimab	Outcome
1^[5]^	55 M	Lung adenocarcinoma	Sintilimab, pemetrexed and carboplatin	44 d after the first cycle	Dermatitis bullosa	Piperacllin-tazobactam, fusidic acid cream, cetirizine and prednisone	Stop	Dead
2^[6]^	64 M	Cancer of the esophagogastric	Sintilimab	5 d after the first cycle	Purpura-like cutaneous vasculitis	Methylprednisolone and diphenhydramine	Stop	Survived
3^[7]^	72 M	Cancer, hepatopulmonary metastases	Sintilimab, pacllitaxel, liposome and carboplatin	23 d after the first cycle	Toxic epodermal necrolysis	Methylprednisolone sodium cuccinate injection and prednisone acetate tablets	Stop	Survived
4^[8]^	55 M	Lymphoma	Sintilimab, gemcitabine and oxaliplatin	11 d after the first cycle	Toxic epodermal necrolysis	Oral cetirizine, methylprednisolone, immunoglobulin, piperacillin sodium-tazobactam and parenteral nutrition	Stop	Survived
5^[9]^	72 F	Gallbladder carcinoma	Sintilimab, anlotinib	2 wk after the first cycle	Toxic epodermal necrolysis	Methylprednisolone, immunoglobulin, albumin, encapsulation, tapering of glucocorticoid and oral nystatin.	Stop	Survived
6^[10]^	67 M	Advanced lung squamous carcinoma	Sintilimab, albumin paclitaxel and cisplatin	32 d after the first cycle	Eczema dermatitis	Methylprednisolone and TCM (Xiaofeng powder)	Stop	Survived
7^[11]^	59 M	Squamous cell-lung carcinoma	Sintilimab and conventional chemotherapy	11 d after the first cycle of postoperative stilinimab therapy	Toxic epidermal necrolysis	Intravenous methylprednisolone, prednisone and Levofloxacin	Stop	Survived
8^[12]^	80s M	Thymic carcinoma	Sintilimab	1 wk after the first cycle	Toxic epidermal necrolysis	Intravenous methylprednisolone, followed by oral prednisone	Stop	Died
9^[13]^	24 M	Renal cell carcinoma	Sintilimab and axitinib	About 2 yr after the first cycle	Bullous Pemphigoid	Methylprednisolone, minocycline and nicotinamide	Stop	Survived
10^[14]^	27 F	Undifferentiated nonkeratinizing carcinoma of the nasopharynx	Sintilimab	After the 4th cycle of Sintilimab	Stevens-Johnson syndrome/Toxic epidermal necrolysis	Methylprednisolone, imipenem and immunoglobulin	Not mentioned	Not mentioned
11^[15]^	70 M	Colorectal cancer, liver metastase	Sitilimab	5 mo after the first cycle	Bullous pemphigoid	Oral methylprednisolone	Restart	Survived
12^[16]^	71 M	Non-small cell cell-lung cancer	Sitilimab, gemcitabine and carboplatin	After the 5th cycle of Sintilimab	Lichenoid dermatitis	Oral traditional Chinese herbal formula-modified Weiling decoction	Stop	Survived
13^[17]^	70 F	Advanced gastric malignancy	Sintilimab, oxaliplatin and tiggio	10 d after the first cycle	Toxic epidermal necrolysis	Intravenous methylprednisolone and immunoglobulin, adalimumab	Stop	Survived
14^[18]^	46 F	Lung adenocarcinoma	Sintilimab and QL1706 injection	2 wk after the first cycle	Aggravated rapidly with depigmentation	No additional treatment	Restart	Survived

The mechanism by which ICIs cause irAEs has not been fully elucidated. Some potential mechanisms include increasing T-cell activity against that are present in tumors and healthy tissue, increasing levels of preexisting autoantibodies, an increase in the level of inflammatory cytokines and others.^[19]^

Tissue cross reactivity can be as an explanation for the relationship between irAEs and ICIs efficacy. Antigen sharing, namely cross reactivity, leads to T-cell-mediated response to not just tumor cells but to healthy cells as well. According to the theory of tissue cross reactivity, some scholars believe that^[3]^ tolerability of ICIs with no or minimal adverse events is potentially indicative of lack of efficacy, so that the development of moderate or severe irAEs can serve as a surrogate marker of response to ICIs. For this reason, the occurrence of adverse reactions is perhaps not a bad thing. But conflicting data are available regarding whether the occurrence of immune-related adverse events is associated with improved treatment efficacy.^[19]^ We need more evidences.

What should we do if a patient presents with adverse effects caused by ICIs? Normally, administration with ICIs be permanently discontinued when severe irAEs happend. A study indicated^[19]^ the safety of retreatment of ICIs probably depends on the severity of the initial immune-related adverse events and retrospective studies have shown that the outcomes for patients whose irAEs were treated with immunosuppression were not worse overall than the outcomes for patients who did not receive immunosuppressive agents for irAEs. Dermatologic adverse events are usually the first to appear and they are usually mild and self-limited. It indicates us that how significant are earlier detection of it and timely intervention. Earlier reporting of symptoms would identify more ircAEs with lower severity which could be treated with timely measures without having to stop ICIs therapy.^[20]^ On the other hand, timely diagnosis and intervention can also prevent progression to serious condition of ircAEs. So patient education is particularly crucial, we should inform patients of a series of irAEs, especially ircAEs that may occur after using ICIS.

For this case, if the local and self-limited rash can be recognized promptly after the first use of Sintilimab as an cutaneous adverse event, with adequate intervention, there may be no recurrence after the second administration, and ultimately the use of Sintilimab would not be withhold.

Last but not least, by reviewing the literature, we found that there have been several clinical trials reporting the use of Sintilimab for colorectal cancer, but it has not yet been included in the drug’s indications. As dermatologists, we have reservations about whether Sintilimab should be used in this patient, and there is no intention in this article to potentially induce non-nonstandard treatment.

## 4. Conclusion

The adverse reactions caused by programmed cell death 1 inhibitors are diverse, and we report here a case of EM drug eruption induced by Sintilimab. In order to use ICIs more safely and efficiently, patient education is also very important. We should inform patients of potential adverse events that may occur after using ICIs. Because skin manifestations are usually the earliest to appear, more special attention should be paid to cutaneous adverse events. Timely identification and intervention can not only prevent the further development of the disease but also may avoid the discontinuation of ICIs.

**Figure 5.**
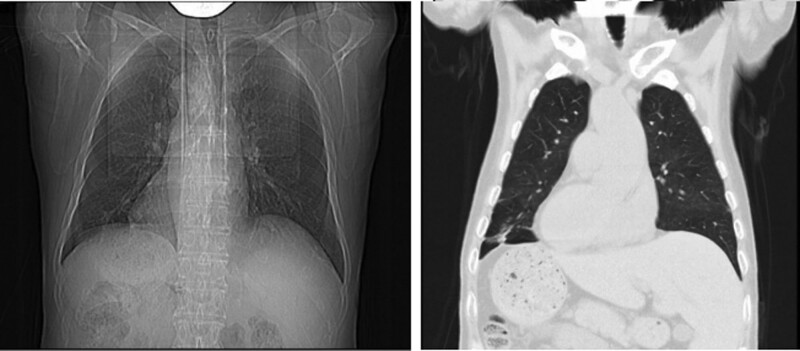
The patient’s chest X-ray and CT showed a mirror-image transposition of both the abdominal and the thoracic organs.

It is worth mentioning that this patient has Situs Inversus Totails (Fig. 5), which is a rare congenital abnormality characterized by a mirror-image transposition of both the abdominal and the thoracic organs.^[21]^

## Acknowledgements

The authors would like to thank the patient who are willing to share his case and his family’s support. The authors would also like to thank the physicians, nurses, research coordinators and other staff at the hospital.

## Author contributions

**Data curation:** Ran Wu, Shaqin Sun.

**Methodology:** Min Jia.

**Resources:** Ting Tang.

**Writing – original draft:** Mei Zhang.

**Writing – review & editing:** Lei Zhang, Ting Tang.
